# Usutu virus, Austria and Hungary, 2010–2016

**DOI:** 10.1038/emi.2017.72

**Published:** 2017-10-11

**Authors:** Tamás Bakonyi, Károly Erdélyi, René Brunthaler, Ádám Dán, Herbert Weissenböck, Norbert Nowotny

**Affiliations:** 1 Viral Zoonoses, Emerging and Vector-Borne Infections Group, Institute of Virology, University of Veterinary Medicine, Veterinaerplatz 1, 1210 Vienna, Austria; 2 Department of Microbiology and Infectious Diseases, University of Veterinary Medicine, Budapest, Hungária krt. 23-25, 1143 Budapest, Hungary; 3 Veterinary Diagnostic Directorate, National Food Chain Safety Office, Tábornok utca 2, 1149 Budapest, Hungary; 4 Institute of Pathology and Forensic Veterinary Medicine, University of Veterinary Medicine, Veterinaerplatz 1, 1210 Vienna, Austria; 5 Department of Basic Medical Sciences, College of Medicine, Mohammed Bin Rashid University of Medicine and Health Sciences, PO Box 505055, Dubai Healthcare City, Dubai, United Arab Emirates

**Keywords:** arbovirus, Austria, blackbird, flavivirus, Hungary, *Turdus merula*, Usutu virus

## Abstract

Usutu virus (USUV, *Flaviviridae*) was first reported in Europe in Austria in 2001, where it caused wild bird (mainly blackbird) mortality until 2005. Since 2006 no further USUV cases were diagnosed in the country. However, the virus emerged in other European countries (Hungary, Italy, Switzerland, Spain, Germany and the Czech Republic) between 2005 and 2011. In 2016, widespread USUV-associated wild bird mortality was observed in Germany, France, Belgium and the Netherlands. In this study, we report the results of passive monitoring for USUV in Austria and Hungary between 2010 and 2016. In Hungary, USUV caused sporadic cases of wild bird mortality between 2010 and 2015 (altogether 18 diagnosed cases), whereas in summer and autumn 2016 the number of cases considerably increased to 12 (ten blackbirds, one Eurasian jay and one starling). In Austria, USUV was identified in two blackbirds in 2016. Phylogenetic analyses of coding-complete genomes and partial regions of the NS5 protein gene revealed that USUVs from Hungary between 2010 and 2015 are closely related to the virus that emerged in Austria in 2001 and in Hungary in 2005, while one Hungarian sequence from 2015 and all sequences from Hungary and Austria from 2016 clustered together with USUV sequences reported from Italy between 2009 and 2010. The results of the study indicate continuous USUV circulation in the region and exchange of USUV strains between Italy, Austria and Hungary.

## INTRODUCTION

In August 2001, an episode of increased wild bird mortality was observed in the eastern part of Austria, mainly in Vienna and its surroundings. Eurasian (or common) blackbirds (*Turdus merula*) and great gray owls (*Strix nebulosa*) were affected. Histopathological investigations revealed non-suppurative encephalitis. A virus was isolated from the brain sample of one blackbird, which was genetically identified as Usutu virus (USUV, *Flaviviridae, Flavivirus*).^1^ USUV RNA was detected in other blackbird and great gray owl samples in 2001 and further cases were diagnosed in 2002.^2^ Between 2003 and 2006 surveillance programs demonstrated consecutive outbreaks of USUV-associated encephalitis and mortalities in the eastern federal states of Austria, however, the number of cases sharply decreased over the years;^3^ simultaneously, the number of wild birds with anti-USUV antibodies markedly increased in the affected populations.^4^ Since 2006, when active monitoring was formally discontinued, no further USUV cases were diagnosed in Austria. Subsequently, the emergence of USUV was reported in other European countries including Hungary in 2005,^5^ Italy in 2006,^6^ Switzerland in 2006,^7^ Spain in 2006,^8, 9^ Germany in 2011,^10^ the Czech Republic in 2011,^11^ Belgium in 2012^12^ and France in 2015.^13^ Serological studies indicated the presence of anti-USUV antibodies in migratory and resident birds in further European countries.^14, 15^

In late summer and early autumn of 2016, widespread USUV activity was observed in several Western European countries including the Netherlands, Belgium, France and Germany.^16, 17, 18^ In this paper, we report the results of our USUV passive surveillance studies in two central European countries, Austria and Hungary, between 2010 and 2016.

## MATERIALS AND METHODS

In Austria, targeted USUV surveillance programs were not implemented since 2006. However, a limited number of songbird carcasses were submitted for investigation by citizens and ornithologists. Altogether seven wild bird carcasses were tested for USUV infection between 2010 and 2015: three birds from one mortality episode in Upper Austria and two single birds from Vienna (2010), a single bird from Upper Austria in 2011, one bird from Styria in 2013 and two birds from Lower Austria in 2014. At the end of September and beginning of October 2016 increased blackbird mortality was reported from the federal state of Lower Austria in areas surrounding the city of Vienna. From this episode, three blackbird carcasses were submitted for USUV investigations, and an additional one from the city of Graz, Styria.

In Hungary, passive surveillance of dead wild birds has been performed each year as part of the avian influenza monitoring program. A selected subset of these bird specimens with the possibility of USUV infection (thrushes and other songbirds, owls, cases from mortality events during the vector season, indicative gross- and histopathological findings) was also tested for the presence of USUV antigen and/or nucleic acid. Between 2010 and 2016, altogether 83 dead birds were investigated for USUV infection (2010: 1, 2011: 3, 2012: 7, 2013: 6, 2014: 10, 2015: 18, and 2016: 38).

The carcasses were necropsied and tissue samples (brain, spleen, liver, heart, kidney, intestine and pancreas) were collected for histopathological and immunohistochemical investigations from 7 Austrian and 22 Hungarian birds in adequate condition as described earlier.^3, 5^ The presence of USUV-specific RNA was tested by end-point RT-PCR and TaqMan real-time RT-PCR methods^19^ in all bird samples submitted for investigations. The coding-complete genome sequence of one Austrian and one Hungarian USUV was determined by Sanger sequencing of overlapping RT-PCR amplification products as previously described.^20^ Sequences were identified by BLAST search (https://blast.ncbi.nlm.nih.gov/Blast.cgi). Phylogenetic and molecular evolutionary analyses of selected USUV coding-complete nucleotide sequences were conducted using neighbor-joining, maximum likelihood, minimum evolution, UPGMA and maximum parsimony algorithms (MEGA version 6, with 1000 replicates for bootstrap testing).

Another phylogenetic analysis was performed using partial nucleotide sequences of the NS5 coding region, between nt positions 9124 and 9308 according to the reference USUV sequence (GenBank accession no. NC_006551). In this analysis further sequences, reported by Cadar *et al.*^17^ and Calzolari *et al.*,^21^ were included. Bayesian analysis was performed employing the Beast2 software package^22^ for 5 million generations, with sampling at every 10th generation, using the HKY+I substitution model, assuming constant population and a strict molecular clock. Obtained trees were processed using 10% burning-in in TreeAnnotator and edited by FigTree software(http://tree.bio.ed.ac.uk/software/figtree/).

## RESULTS

In Austria, all seven wild bird samples submitted between 2010 and 2015 were tested negative for USUV. Of the three carcasses from an episode of blackbird mortality in Lower Austria submitted in fall 2016, USUV infection was diagnosed in two of them by immunohistochemistry (IHC) and RT-PCR tests. The positive blackbirds were found on 29th September and 3rd October and they originated from Fischamend (48°7′N, 16°37′E) and from Königstetten (48°17′N, 16°7′E), respectively. The third blackbird from Lower Austria and the one from Graz tested USUV-negative.

**Figure 1:**
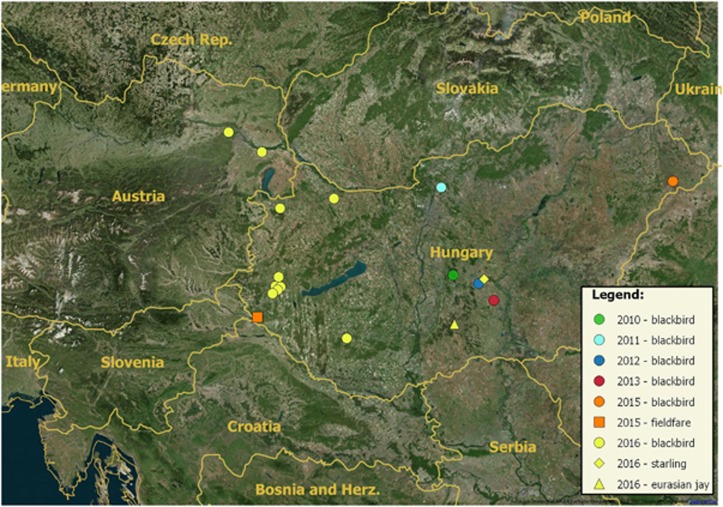
Map indicating the locations where USUV-positive birds were found in Hungary and in Austria between 2010 and 2016. Years are indicated with color codes (2010: green, 2011: cyan, 2012: blue, 2013: red, 2015: orange, 2016: yellow). Host species are indicated with symbols (circle: blackbird, triangle: Eurasian jay, diamond: starling, square: fieldfare).

In Hungary, USUV was detected between 2010 and 2015 in 18 birds, first from the central and then from the western and eastern part of the country (2010: one blackbird from Kunadacs (46°57′N, 19°17′E), 2011: one blackbird from Vác (47°48′N, 19°08′E), 2012: one blackbird from Kecskemét (46°54′N, 19°39′E), 2013: one blackbird from Kiskunfélegyháza (46°71′N, 19°51′E), 2015 one blackbird from Fábiánháza (47°51′N, 22°22′E) and one fieldfare (*Turdus pilaris*) from Lovászi (46°33′N, 16°33′E)). In 2016, USUV was diagnosed in one Eurasian jay (*Garrulus glandarius)* from Imrehegy (46°27′N, 19°18′E), one starling (*Sturnus vulgaris*) from Kecskemét (46°54′N, 19°39′E) and in ten blackbirds. The first blackbird carcass was submitted on 29th July from Kaposvár (46°24′N, 17°48′E), followed by cases from Győr (47°41′N, 17°37′E) and Fertőszentmiklós (47°34′N, 16°52′E) on 16th September, and a cluster of blackbird mortality cases was reported between 26th September and 15th October from Zala county (Zalaegerszeg (46°50′N, 16°50′E), Nagylengyel (46°46′N, 16°45′E) and Egervár (46°56′N, 16°50′E)). The geographic distribution of the positive cases is shown in Figure 1.

The birds subjected to detailed pathological examination were in good post-mortem condition or moderately autolytic. The nutritional status was mildly emaciated. Gross lesions observed in both cases were moderate to severe splenomegaly, mild hepatomegaly and general congestion of internal organs.

The histopathological examination revealed moderate multifocal hepatic necrosis, partly accompanied by inflammatory reaction. The spleen was slightly hyperemic with hyperplasia of the red pulp and focal necrosis. Kidneys showed mild and focal perivascular lymphohistiocytic infiltrations. Immunohistochemistry revealed presence of viral antigen in brain, heart, lung, spleen, intestine and pancreas. In the brain, groups of cortical neurons as well as individual Purkinje cells were positive. In the heart numerous cardiomyocytes and some interstitial cells showed positive reactions. Lung sections also contained single positive cells. In the spleen, some positive signals were present in scattered cells of the splenic pulp and the splenic capsule. A positive reaction was also found in crypt epithelia and connective tissue cells of the intestine as well as multifocally in the pancreas. The observed gross lesions, histopathological and IHC findings corresponded to our previous observations.^1, 5, 6, 23^ For pictures, we therefore kindly refer to these papers.

**Figure 2:**
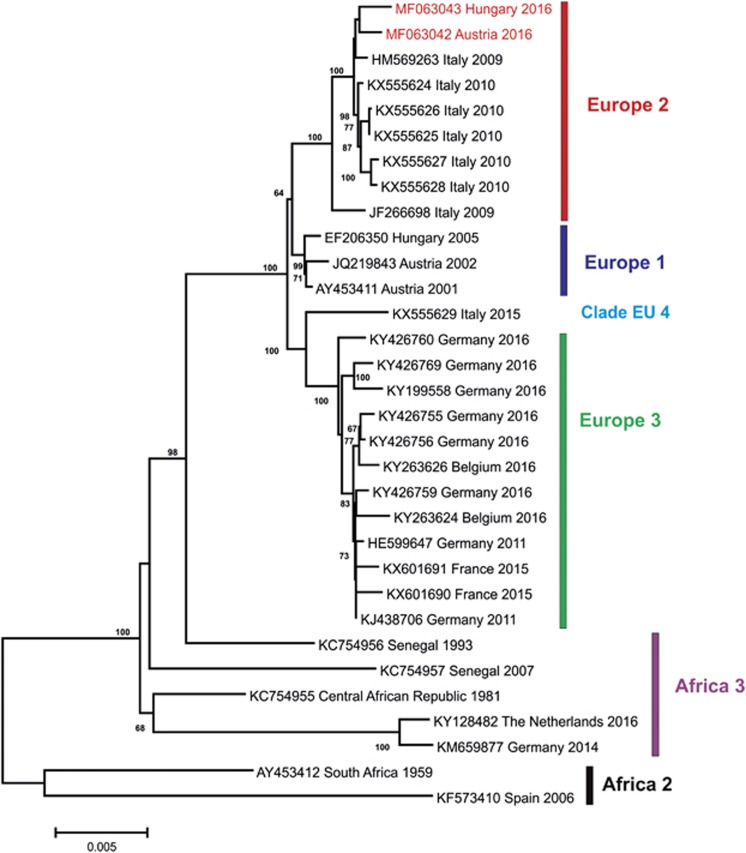
Phylogram demonstrating the genetic relationships among Usutu viruses based on coding-complete nucleotide sequences. Sequences are labeled by codes containing the GenBank accession number, country of origin, and year of sample collection. The sequences from Austria and Hungary described in this paper are highlighted in red font. Vertical bars on the right indicate USUV genetic lineages (as suggested by Cadar *et al.*^17^ and Calzolari *et al.*^21^). The phylogram was generated with neighbor-joining statistical method; bootstrap percentage values of 1000 replicates above 60% are displayed. Horizontal bar on the left represents the genetic distance.

The genomes of two USUVs from 2016 (one from Fischamend, Austria and one from Zalaegerszeg, Hungary) were amplified by endpoint RT-PCRs resulting in overlapping DNA products. Sequences were aligned to the reference USUV sequence and were compiled to continuous sequences. The genome sequence of the Blackbird-Fischamend-2016 USUV was determined between nucleotide (nt) positions 5 and 11 066, according to the reference sequence; the sequence of the Blackbird-Zalaegerszeg-2016 USUV was determined between nt positions 10 and 10 854. The sequences were deposited in GenBank database under accession numbers MF063042 and MF063043, respectively. Despite repeated attempts with different primer pair combinations, an about 210-nt-long stretch at the 3′-end untranslated region (UTR) of the Blackbird-Zalaegerszeg-2016 USUV could not be amplified and sequenced. However, the open reading frame between nt positions 97 and 10 401 was determined for both viruses and the sequences were translated to 3434 amino-acid long sequences of the putative polyprotein precursor. Multiple nucleotide sequence alignments were generated with the newly determined two sequences and USUV complete genome sequences available in public databases. Representative sequences of the suggested USUV genetic lineages ‘Europe 1’ to ‘Europe 3’, ‘Africa 2’ and ‘Africa 3’ were included.^17^ Unfortunately, only partial nucleotide sequences are available for USUVs belonging to the proposed ‘Europe 4’, ‘Europe 5’ and ‘Africa 1’ genetic lineages; therefore, these viruses could not be included in this analysis. The inferred genetic relationships are shown in a phylogram (Figure 2). The nucleotide sequences of USUVs detected in blackbirds in Austria and Hungary in 2016 cluster together with Italian sequences identified in a human plasma sample and in blackbirds between 2009 and 2010. These sequences represent the suggested ‘Europe 2’ genetic lineage of USUV. The USUV sequences from Austria and Hungary determined between 2001 and 2005 cluster in a neighboring but distinct branch (Europe 1). Phylogenetic analyses with different statistical algorithms resulted in very similar tree topologies.

**Figure 3:**
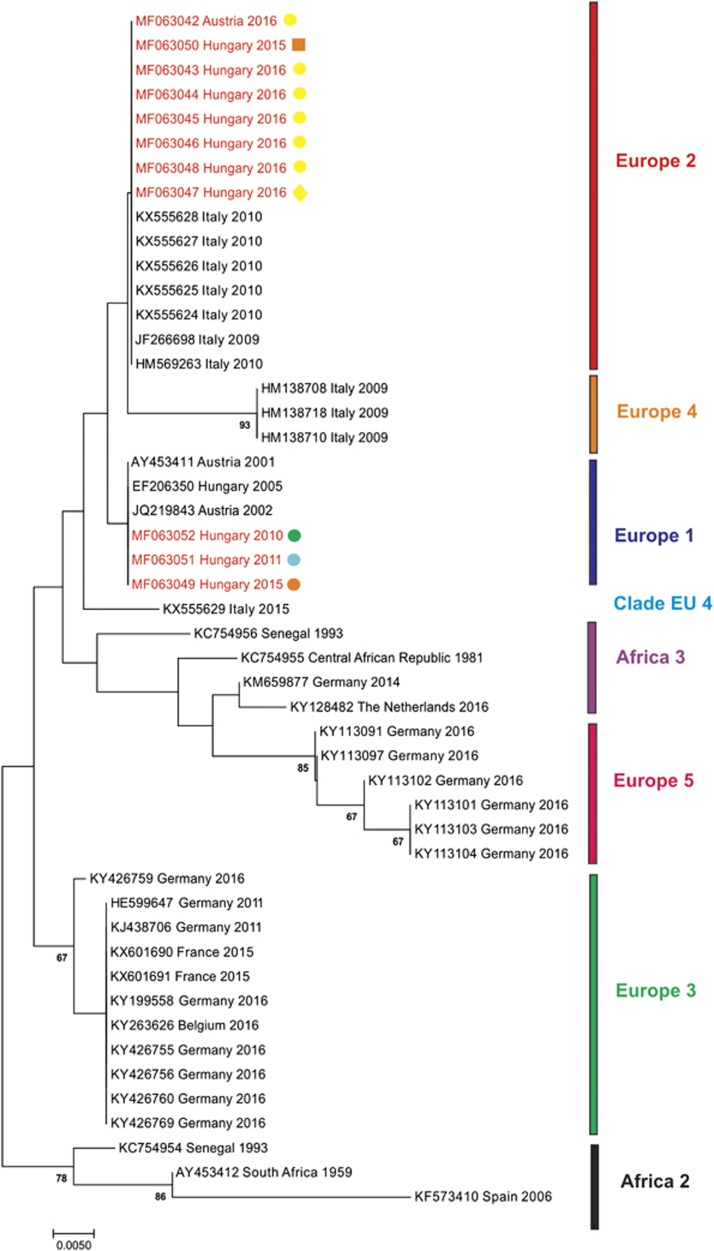
Phylogram demonstrating the genetic relationships among Usutu viruses based on partial NS5 protein coding nucleotide sequences. Sequences are labeled by codes containing the GenBank accession number, country of origin and year of sample collection. The sequences from Austria and Hungary described in this paper are highlighted in red font. Colored symbols indicate host species and year of collection (corresponding with Figure 1). Vertical bars on the right indicate USUV genetic lineages (as suggested by Cadar *et al.*^17^ and Calzolari *et al.*^21^). The phylogram was generated with Bayesian statistical method; posterior support values above 60% are displayed. Horizontal bar on the left represents the genetic distance.

In order to include also those currently suggested USUV genetic lineages, for which only partial sequences are available, a phylogenetic analysis of a partial NS5 gene region was performed. Besides the sequences used in the phylogenetic analysis of the coding-complete genomes (Figure 2), further sequences from Hungarian birds from 2010, 2011, 2015 and 2016 (GenBank accession numbers MF063044–MF063052) and representatives of the ‘Europe 4’ and ‘Europe 5’ genetic lineages suggested by Cadar *et al.*^17^ and clade ‘Europe 4’ suggested by Calzolari *et al.*^21^ were included in the partial NS5 phylogram (Figure 3). The Austrian and Hungarian sequences from 2016 and one sequence from Hungary from 2015 cluster together with sequences from Italy collected in 2009 and 2010 (lineage ‘Europe 2’). However, three USUV sequences from Hungary collected in 2010, 2011 and 2015, respectively, grouped together with the ‘old’ sequences from Austria (collected in 2001 and 2002) and Hungary (collected in 2005) (lineage ‘Europe 1’). However, due to the shortness of sequences, certain nodes obtained only poor statistical support.

## DISCUSSION

Fluctuation in arbovirus activity is a frequently seen phenomenon.^24^ The spatio-temporal dynamics of a given arbovirus can be influenced by several factors. In temperate climate zones, seasonality may fundamentally influence vector activity. Variance in weather conditions has major effects on vector abundance and the extrinsic incubation period of arboviruses.^25^ The inter-annual patterns of arbovirus activity, however, are further affected by host factors (that is, abundance of susceptible vertebrate hosts, herd immunity, and so on). Taking these factors into consideration, a statistical model was developed to describe and predict USUV dynamics.^26^

The circulation of USUV in sylvatic habitats usually remains obscure. This may explain why in Africa USUV has been detected most frequently accidentally, as results of arbovirus surveillances in mosquitoes.^27^ In Europe, outbreaks of USUV-associated bird mortalities were typically reported in urban habitats, affecting mainly blackbirds or different owl species kept in captivity. In such situations, public awareness significantly helps in recognizing the increased mortality, and citizens then submit dead birds for diagnostic investigations.^2^ The first USUV outbreak in Germany in 2011 was also recorded in urban environments.^28^ However, in Northern Italy, where integrated and consistent mosquito and wild bird monitoring programs with focus on West Nile virus (WNV) and USUV are in place, the presence of USUV was confirmed each year between 2008 and 2015.^21, 29^

In Hungary, regular USUV activity was detected from 2010 to 2013 in the central and eastern region, while during 2015 and 2016 circulation of the virus was also evidenced in the western part of the country. The fieldfare case detected in 2015 occurred in Zala county close to the Slovenian border; it may be considered the precursor of the 2016 outbreak since the detected USUV strain was identified as a member of the ‘Europe 2’ lineage based on its partial NS5 sequence. These data may indicate that USUV is continuously circulating in certain areas without obvious or too conspicuous wild bird mortality. Monitoring of flavivirus-related bird mortality events also revealed the continuous presence and circulation of USUV strains belonging to the ‘Europe 1’ lineage. These virus strains were identified in individual blackbird cases in 2010, 2011 and 2015.

In Austria, 10 years after the last diagnosed case, USUV ‘re-emerged’ and caused again blackbird mortality (reported by citizens of Lower Austria). Although a low number of carcasses was submitted for investigations, the infection and associated lesions were detected in two blackbirds.

In Hungary altogether 12 birds tested positive for USUV infection in 2016, which is the highest annual number ever diagnosed in the country. Weather conditions may have contributed to the increased virus activity. For example, the mean temperature in September was 2 °C higher than the long-term mean (1980–2010), and the precipitation in July exceeded the 30 years mean by 96%.^30^

The origin, evolutionary history and spread of USUV in Europe was recently analyzed in different studies.^17, 21, 31^ Phylogenetic analyses revealed that the so-far discovered USUVs form at least six clusters (‘genetic lineages’) that mainly correspond with the geographic origins of the strains (genetic lineages ‘Africa 1’ to ‘Africa 3’ and ‘Europe 1’ to ‘Europe 3’).^31, 32^ However, analysis of the nucleotide sequence diversity of USUVs detected in Germany in 2016 revealed further clustering, therefore in the publication of Cadar *et al.*^17^ five European genetic lineages (‘Europe 1’ to ‘Europe 5’) were suggested (by splitting the former ‘Europe 2’ cluster into ‘Europe 2’ and ‘Europe 4’ and the establishment of a ‘Europe 5’ lineage.) The phylogenetic analysis by Calzolari *et al.*^21^ revealed four ‘European clades’: ‘Europe 1’ to ‘Europe 3’ are practically identical with the ‘lineages’ suggested by the previous studies, but ‘Europe 4’ contains USUV sequences of Italian origin, which were not included in the analysis by Cadar *et al.*^17^ Vice versa, USUV sequences clustering into the suggested genetic lineages ‘Europe 4’ and ‘Europe 5’^17^ were not included in the analysis by Calzolari *et al.*^21^ This might result in some confusion in scientific communications, particularly because on-going research activities in the field will presumably discover several further genetic variants. A consensus nomenclature for the genetic clusters of USUV would therefore be very useful. Anyway, according to the International Committee on Taxonomy of Viruses^33^ groupings below species level have no formally accepted taxonomic meaning. It is also worth mentioning that several USUVs detected in Europe do not cluster within ‘European lineages’, but are part of the ‘Africa 2’ and ‘Africa 3’ groups;^17^ therefore such geographical distinctions are not entirely justified although it is understandable to label genetic varieties with certain names. The aforementioned phylogenetic studies used partial nucleotide sequences of the E and NS5 protein coding regions, and Bayesian and/or maximum-likelihood statistical algorithms were applied. The probable spread of the viruses was also modeled, indicating a primary introduction of USUV from Africa into Austria and subsequent spread of the virus to Hungary, Northern Italy, Switzerland, Germany and the Czech Republic.^21, 31^

In the present study, the coding-complete nucleotide sequences of USUVs detected in 2016 in Austria and in Hungary were analyzed. Phylogenetic comparisons with the available USUV complete genome sequences revealed that the Austrian and Hungarian sequences form a monophyletic cluster together with USUV sequences detected in Italy between 2009 and 2010 (100% bootstrap support). This cluster corresponds to the ‘Europe 2 genetic lineage’ and it is distinct from the USUVs that emerged in Austria in 2001 and in Hungary in 2005 (‘Europe 1’). These results indicate a transboundary exchange of USUVs between Northern Italy, Austria and Hungary. Several songbirds in central Europe are short-distant migrants, including about 20% of the blackbird population resident in the Carpathian basin that overwinters in the Mediterranean Basin (mainly Central and Northern Italy).^34, 35^ These birds may contribute to the dispersal of USUV through their flyways. In Germany and other Western European countries, these bird species use a distinct migratory route at the western side of the Alps, which may provide a reasonable explanation for the genetic separation of the dominant USUVs in those regions. However, the detection of representatives of other genetic clusters in Western Europe^17, 31, 36^ indicates that the genetic flow is more extensive in Europe, for example, the recent (2015–2016) sequences from Germany, Belgium and France form another distinct cluster (‘Europe 3’), which also contains sequences from Germany (2011) and a sequence from Italy (from 2015). The sequences of the suggested genetic lineages ‘Africa 2’ and ‘Africa 3’ show much higher sequence diversity/longer evolutionary distances than the European lineages, and the suggested ‘Africa 3’ lineage most likely consists of sequences of polyphyletic origins including sequences from Germany 2014 (strain ‘Bonn’, derived from a blackbird) and the Netherlands 2016 (derived from a great gray owl).

The phylogenetic analysis of partial NS5 gene sequences revealed similar clustering of the sequences compared to the coding-complete sequence-based inference in respect to the Austrian and Hungarian sequences. USUV sequences detected in blackbirds in Hungary in 2010, 2011 and 2015 cluster in lineage ‘Europe 1’, while another sequence from Hungary from 2015 and all sequences from 2016 belong to the ‘Europe 2’ lineage. However, the analysis based on the partial NS5 sequences provided evolutionary inferences with poor statistical supports, and some (European and African) sequences are clustering differently in the coding-complete and partial NS5 sequence-based trees. Determination of complete nucleotide sequences of the various USUV strains available at the different research groups could provide a more solid basis for establishing evolutionary scenarios and for developing spatiotemporal models.

The previously mentioned, spatiotemporal models hypothesize primary introduction of USUV to Austria and subsequent local spread. However, a sampling bias may constitute the basis of these hypotheses, namely that USUV was first diagnosed in Austria in 2001, and the oldest European USUV complete nucleotide sequence was determined from this outbreak.^20^ Additionally, a retrospective study provided evidence for USUV-associated wild bird mortality in 1996 in the Tuscany region of Italy.^19^ Because only formalin-fixed, paraffin-embedded tissue samples were available for investigation, only short nucleotide sequences (158 nt) of the NS5 region could be revealed. They were 100% identical to the corresponding sequence of the first Austrian isolate (AY453411), and 98.7%–99.3% identical to other central European USUV sequences.^19^ If we take into consideration that USUV has been continuously circulating in Northern Italy^21^ and there is a transboundary USUV exchange between Italy and Austria (as the results of this study indicate), a slightly different scenario of initial introduction of USUV from Africa to Europe should also be considered.^19^ Further studies may reveal ecological factors as driving forces for the establishment and survival of ‘dominant’ genetic clusters in different geographic areas.

Interestingly, across all genetic variants, blackbirds and great gray owls remain to be the most vulnerable avian species for USUV infections.^1, 16^ Besides being pathogenic for certain bird species, USUV has been recognized as the causative agent of human encephalitis.^37, 38, 39, 40^ In addition, USUV RNA or anti-USUV antibodies were detected in clinically healthy people including blood donors in Europe.^41, 42^ A retrospective study reported in the area of the municipality of Modena, Italy, even more USUV RNA and antibody positives among human encephalitic cases than WNV positives.^43^ Considering its zoonotic potential and challenges in WNV infection diagnostics (due to antigenic cross reactivity),^44^ further emphasis should be put on monitoring and epidemiological analyses of USUV in Europe.
